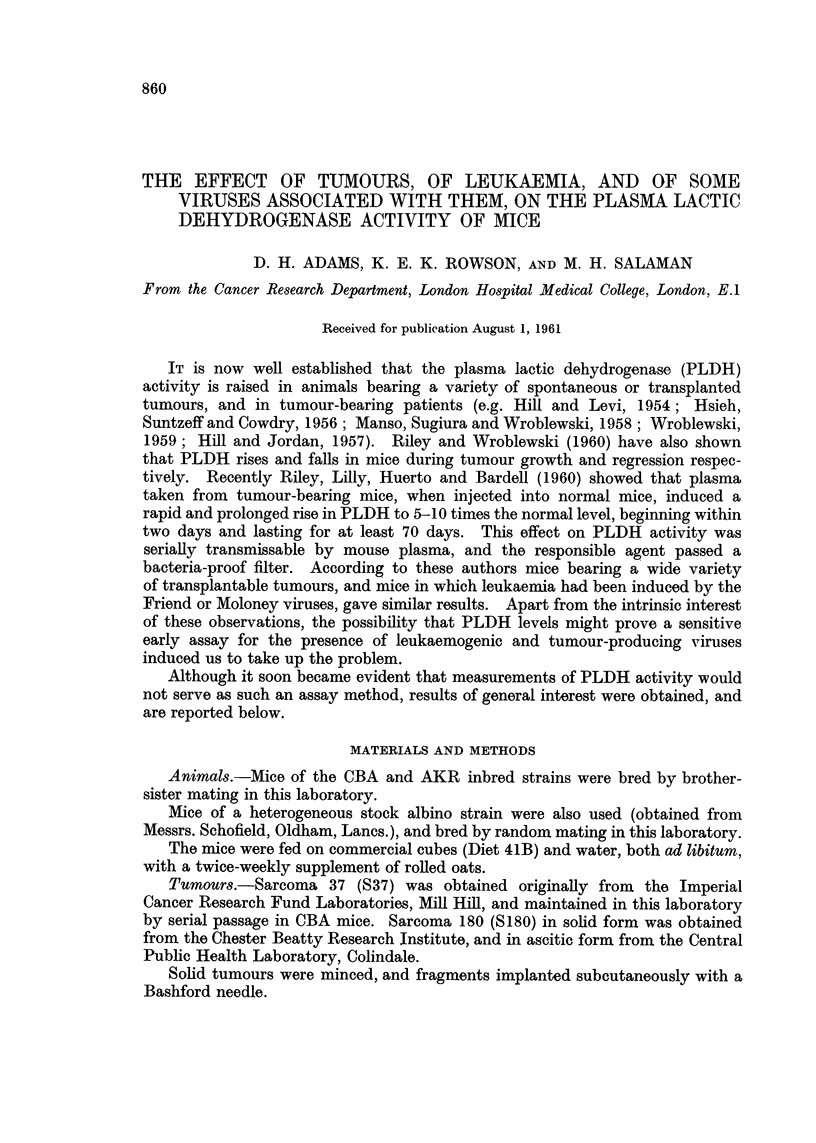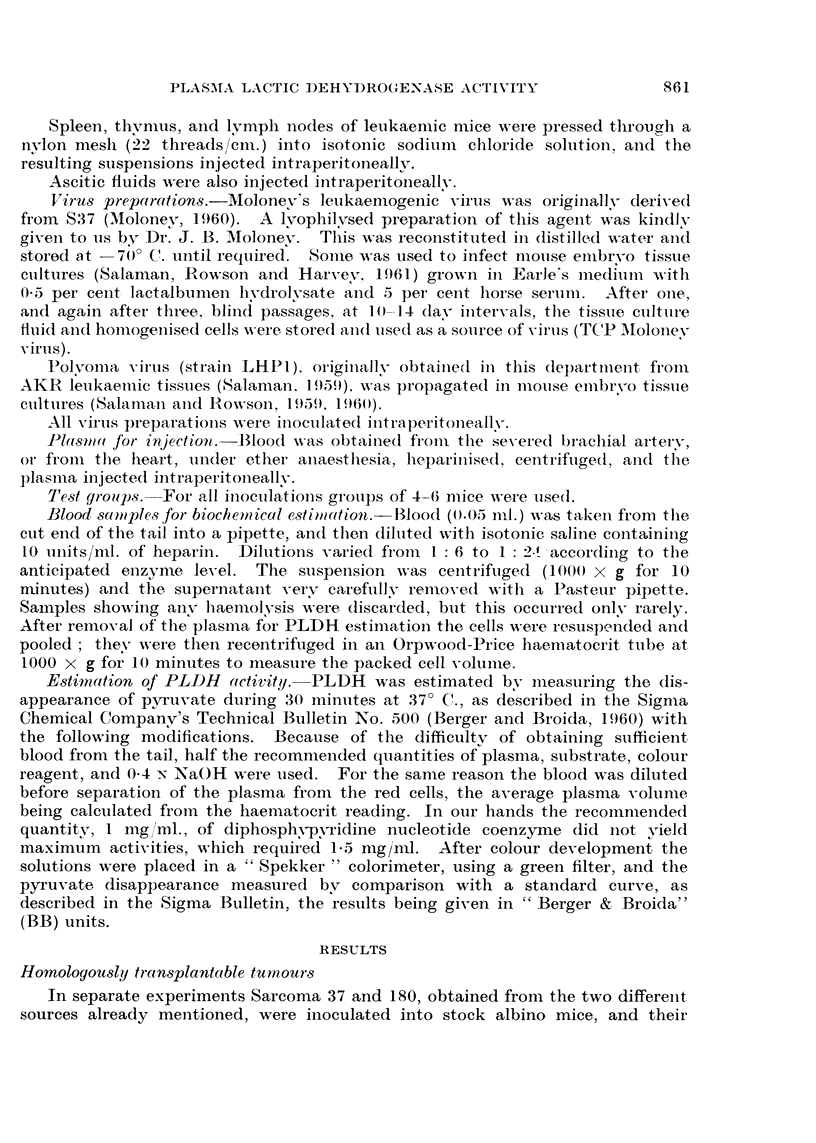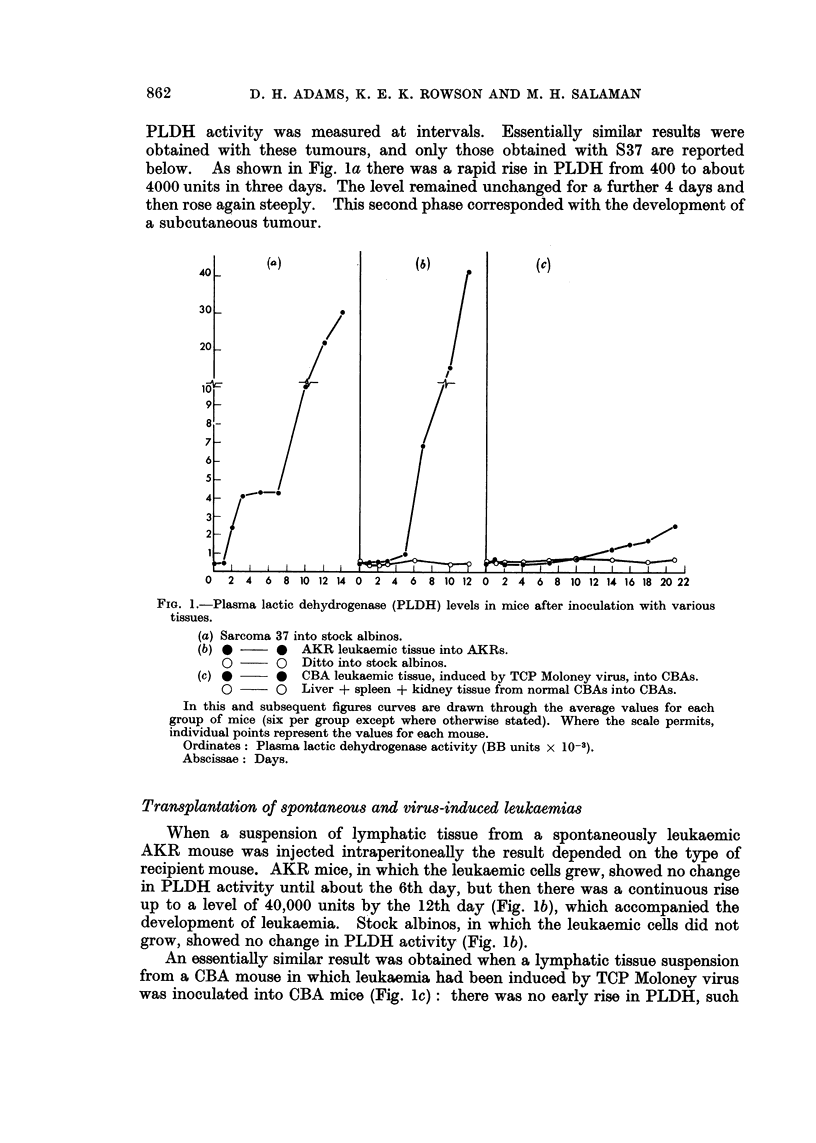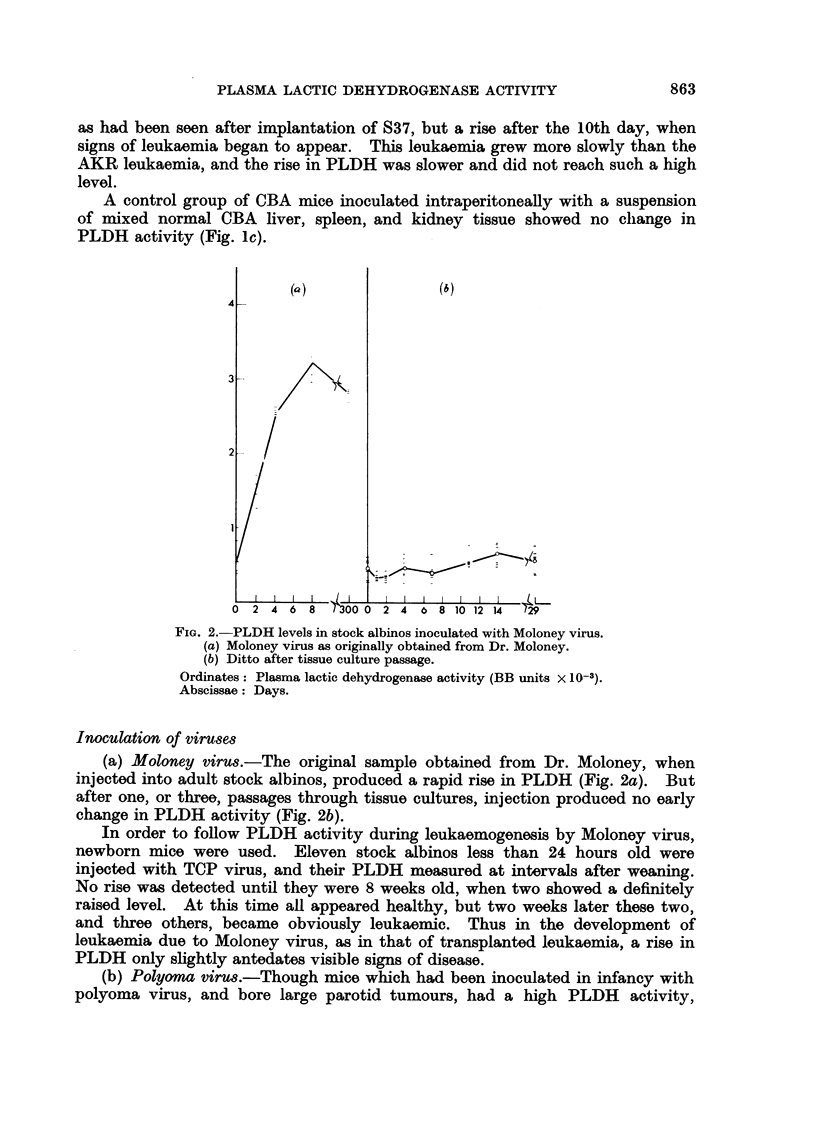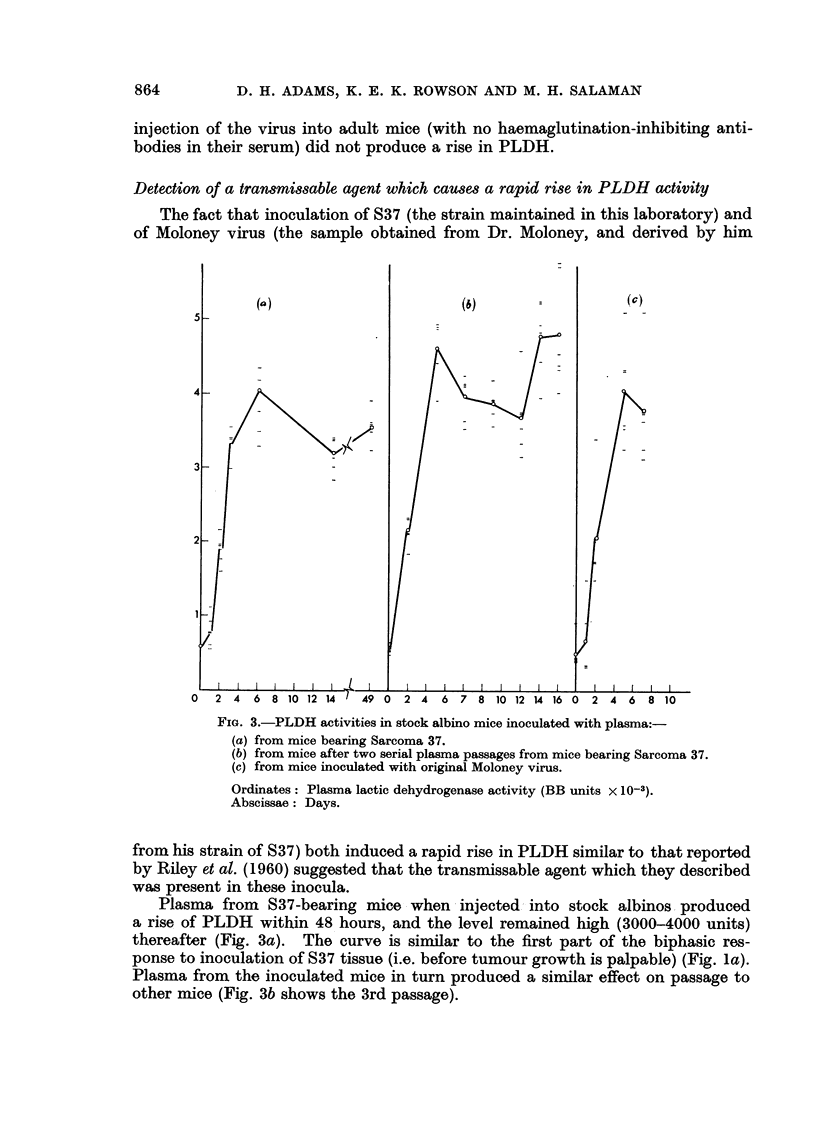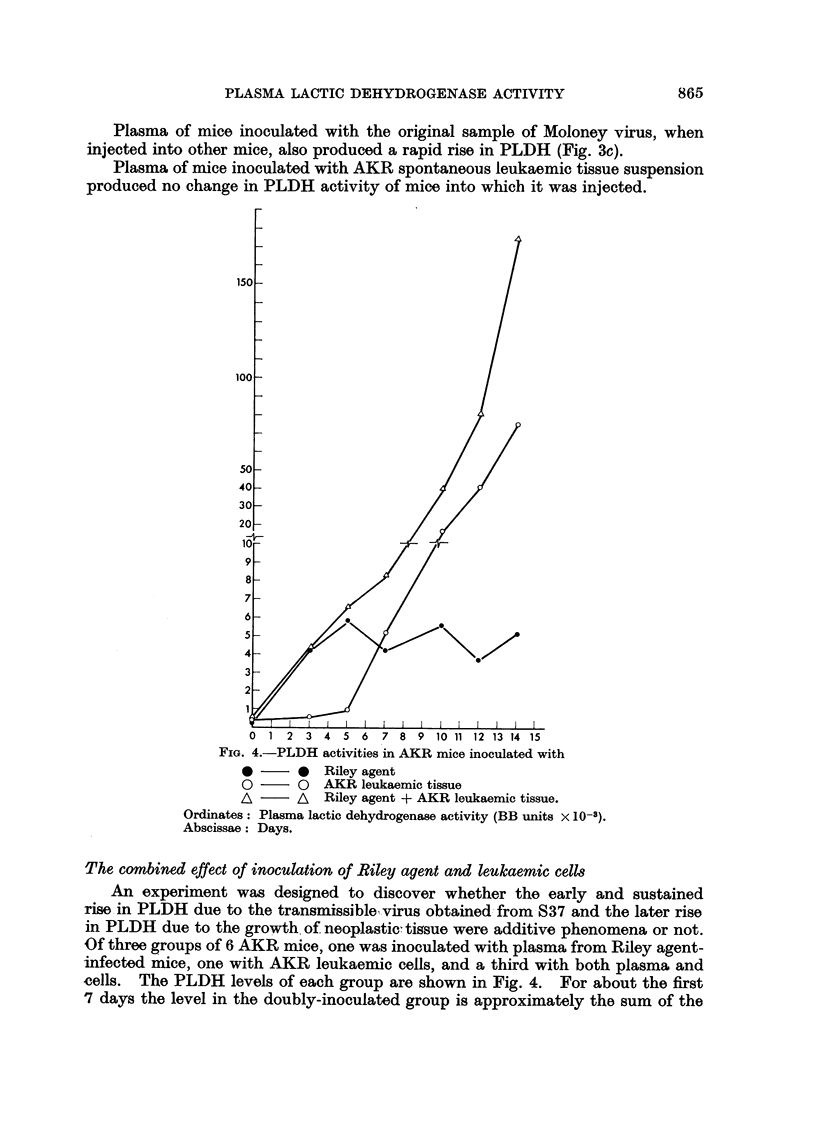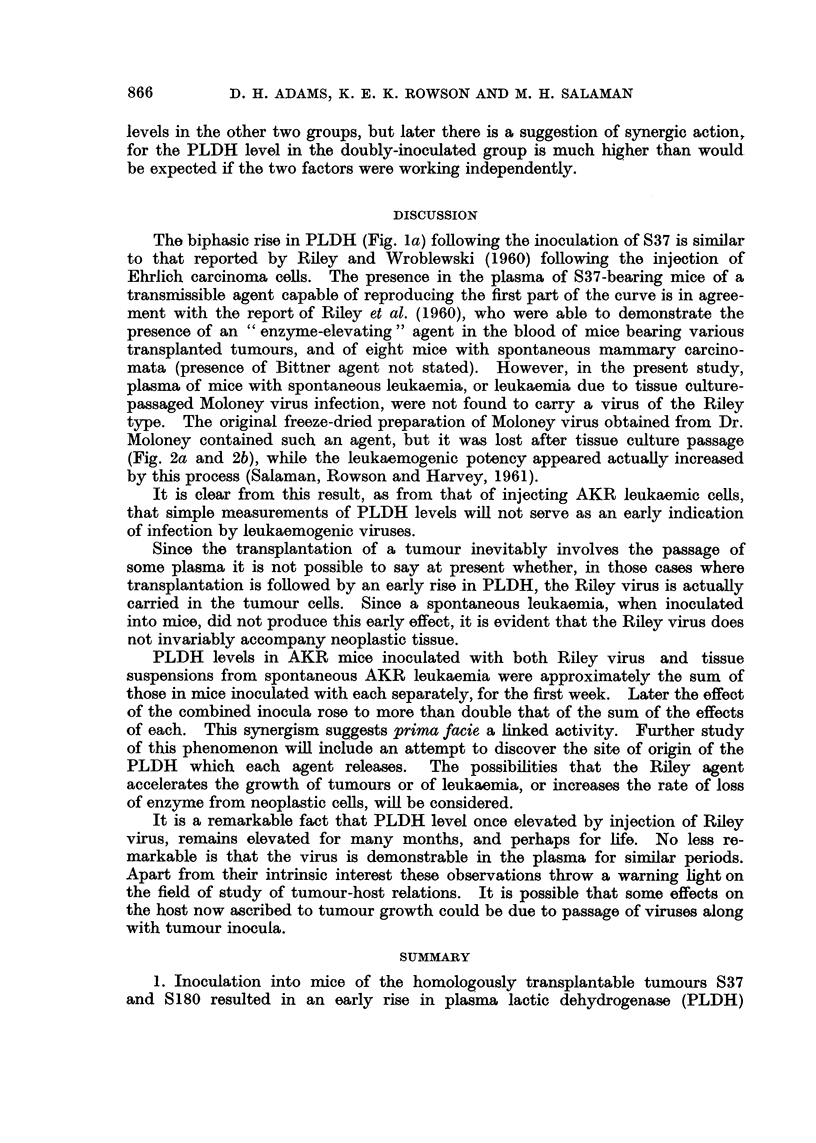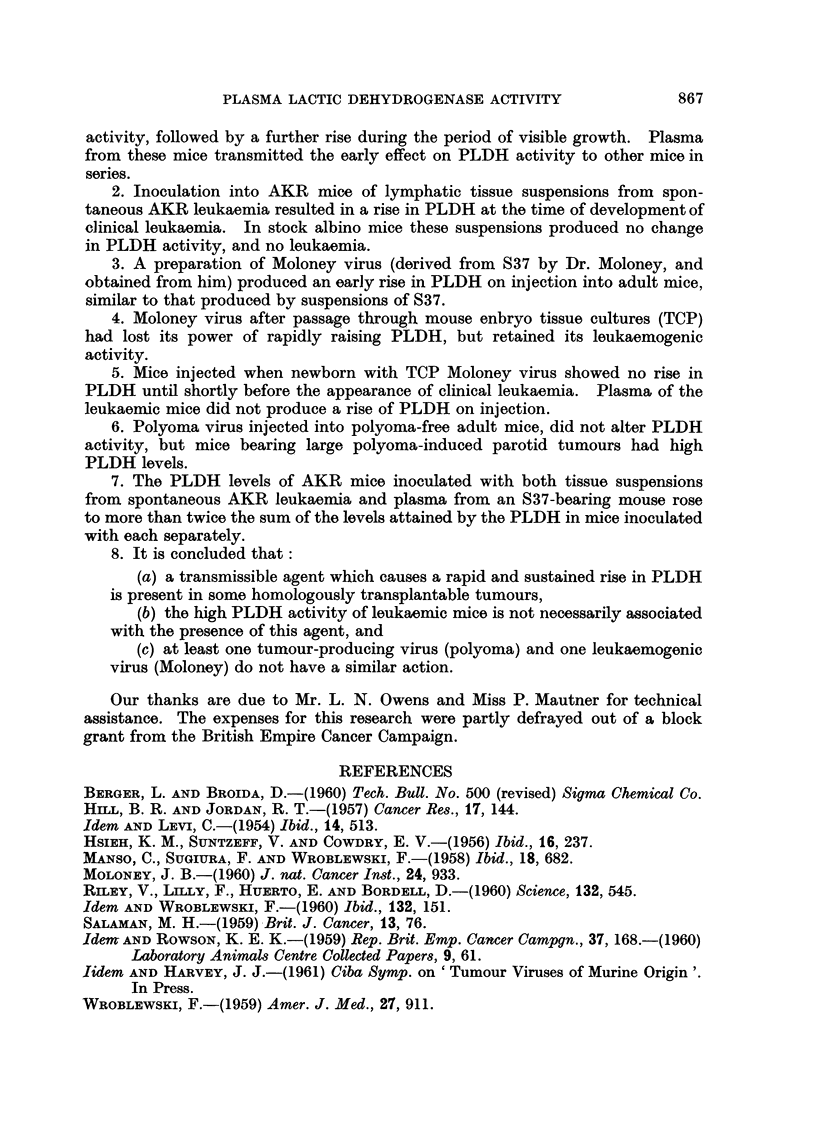# The Effect of Tumours, of Leukaemia, and of Some Viruses Associated with them, on the Plasma Lactic Dehydrogenase Activity of Mice

**DOI:** 10.1038/bjc.1961.100

**Published:** 1961-12

**Authors:** D. H. Adams, K. E. K. Rowson, M. H. Salaman


### THE EFFECT OF TUMOURS, OF LEUKAEMIA, AND OF SOME VIRUSES ASSOCIATED WITH THEM, ON THE PLASMA LACTIC DEHYDROGENASE ACTIVITY OF MICE

D. H. ADAMS, K. E. K. ROWSON, AND M. H. SALAMAN

*From the Cancer Research Department, London Hospital Medical College, London, E.1*

Received for publication August 1, 1961

It is now well established that the plasma lactic dehydrogenase (PLDH) activity is raised in animals bearing a variety of spontaneous or transplanted tumours, and in tumour-bearing patients (e.g. Hill and Levi, 1954; Hsieh, Suntzeff and Cowdry, 1956; Manso, Sugiura and Wroblewski, 1958; Wroblewski, 1959; Hill and Jordan, 1957). Riley and Wroblewski (1960) have also shown that PLDH rises and falls in mice during tumour growth and regression respectively. Recently Riley, Lilly, Huerto and Bardell (1960) showed that plasma taken from tumour-bearing mice, when injected into normal mice, induced a rapid and prolonged rise in PLDH to 5–10 times the normal level, beginning within two days and lasting for at least 70 days. This effect on PLDH activity was serially transmissable by mouse plasma, and the responsible agent passed a bacteria-proof filter. According to these authors mice bearing a wide variety of transplantable tumours, and mice in which leukaemia had been induced by the Friend or Moloney viruses, gave similar results. Apart from the intrinsic interest of these observations, the possibility that PLDH levels might prove a sensitive early assay for the presence of leukaemogenic and tumour-producing viruses induced us to take up the problem.

Although it soon became evident that measurements of PLDH activity would not serve as such an assay method, results of general interest were obtained, and are reported below.

### MATERIALS AND METHODS

*Animals.*—Mice of the CBA and AKR inbred strains were bred by brother-sister mating in this laboratory.

Mice of a heterogeneous stock albino strain were also used (obtained from Messrs. Schofield, Oldham, Lancs.), and bred by random mating in this laboratory.

The mice were fed on commercial cubes (Diet 41B) and water, both *ad libitum*, with a twice-weekly supplement of rolled oats.

*Tumours.*—Sarcoma 37 (S37) was obtained originally from the Imperial Cancer Research Fund Laboratories, Mill Hill, and maintained in this laboratory by serial passage in CBA mice. Sarcoma 180 (S180) in solid form was obtained from the Chester Beatty Research Institute, and in ascitic form from the Central Public Health Laboratory, Colindale.

Solid tumours were minced, and fragments implanted subcutaneously with a Bashford needle.

Spleen, thymus, and lymph nodes of leukaemic mice were pressed through a nylon mesh (22 threads/cm.) into isotonic sodium chloride solution, and the resulting suspensions injected intraperitoneally.

Ascitic fluids were also injected intraperitoneally.

*Virus preparations.*—Moloney's leukaemogenic virus was originally derived from S37 (Moloney, 1960). A lyophilysed preparation of this agent was kindly given to us by Dr. J. B. Moloney. This was reconstituted in distilled water and stored at $-70°$ C. until required. Some was used to infect mouse embryo tissue cultures (Salaman, Rowson and Harvey, 1961) grown in Earle's medium with 0·5 per cent lactalbumen hydrolysate and 5 per cent horse serum. After one, and again after three, blind passages, at 10–14 day intervals, the tissue culture fluid and homogenised cells were stored and used as a source of virus (TCP Moloney virus).

Polyoma virus (strain LHP1), originally obtained in this department from AKR leukaemic tissues (Salaman, 1959), was propagated in mouse embryo tissue cultures (Salaman and Rowson, 1959, 1960).

All virus preparations were inoculated intraperitoneally.

*Plasma for injection.*—Blood was obtained from the severed brachial artery, or from the heart, under ether anaesthesia, heparinised, centrifuged, and the plasma injected intraperitoneally.

*Test groups.*—For all inoculations groups of 4–6 mice were used.

*Blood samples for biochemical estimation.*—Blood (0·05 ml.) was taken from the cut end of the tail into a pipette, and then diluted with isotonic saline containing 10 units/ml. of heparin. Dilutions varied from 1 : 6 to 1 : 24 according to the anticipated enzyme level. The suspension was centrifuged ($1000 \times$ **g** for 10 minutes) and the supernatant very carefully removed with a Pasteur pipette. Samples showing any haemolysis were discarded, but this occurred only rarely. After removal of the plasma for PLDH estimation the cells were resuspended and pooled ; they were then recentrifuged in an Orpwood-Price haematocrit tube at $1000 \times$ **g** for 10 minutes to measure the packed cell volume.

*Estimation of PLDH activity.*—PLDH was estimated by measuring the disappearance of pyruvate during 30 minutes at 37° C., as described in the Sigma Chemical Company's Technical Bulletin No. 500 (Berger and Broida, 1960) with the following modifications. Because of the difficulty of obtaining sufficient blood from the tail, half the recommended quantities of plasma, substrate, colour reagent, and 0·4 N NaOH were used. For the same reason the blood was diluted before separation of the plasma from the red cells, the average plasma volume being calculated from the haematocrit reading. In our hands the recommended quantity, 1 mg/ml., of diphosphypyridine nucleotide coenzyme did not yield maximum activities, which required 1·5 mg/ml. After colour development the solutions were placed in a " Spekker " colorimeter, using a green filter, and the pyruvate disappearance measured by comparison with a standard curve, as described in the Sigma Bulletin, the results being given in " Berger & Broida" (BB) units.

### RESULTS

### *Homologously transplantable tumours*

In separate experiments Sarcoma 37 and 180, obtained from the two different sources already mentioned, were inoculated into stock albino mice, and their

PLDH activity was measured at intervals. Essentially similar results were obtained with these tumours, and only those obtained with S37 are reported below. As shown in Fig. 1*a* there was a rapid rise in PLDH from 400 to about 4000 units in three days. The level remained unchanged for a further 4 days and then rose again steeply. This second phase corresponded with the development of a subcutaneous tumour.

FIG. 1.—Plasma lactic dehydrogenase (PLDH) levels in mice after inoculation with various tissues.

(*a*) Sarcoma 37 into stock albinos.
(*b*) ● —— ● AKR leukaemic tissue into AKRs.
○ —— ○ Ditto into stock albinos.
(*c*) ● —— ● CBA leukaemic tissue, induced by TCP Moloney virus, into CBAs.
○ —— ○ Liver + spleen + kidney tissue from normal CBAs into CBAs.

In this and subsequent figures curves are drawn through the average values for each group of mice (six per group except where otherwise stated). Where the scale permits, individual points represent the values for each mouse.
Ordinates : Plasma lactic dehydrogenase activity (BB units × $10^{-3}$).
Abscissae : Days.

*Transplantation of spontaneous and virus-induced leukaemias*

When a suspension of lymphatic tissue from a spontaneously leukaemic AKR mouse was injected intraperitoneally the result depended on the type of recipient mouse. AKR mice, in which the leukaemic cells grew, showed no change in PLDH activity until about the 6th day, but then there was a continuous rise up to a level of 40,000 units by the 12th day (Fig. 1*b*), which accompanied the development of leukaemia. Stock albinos, in which the leukaemic cells did not grow, showed no change in PLDH activity (Fig. 1*b*).

An essentially similar result was obtained when a lymphatic tissue suspension from a CBA mouse in which leukaemia had been induced by TCP Moloney virus was inoculated into CBA mice (Fig. 1*c*) : there was no early rise in PLDH, such as had been seen after implantation of S37, but a rise after the 10th day, when signs of leukaemia began to appear. This leukaemia grew more slowly than the AKR leukaemia, and the rise in PLDH was slower and did not reach such a high level.

A control group of CBA mice inoculated intraperitoneally with a suspension of mixed normal CBA liver, spleen, and kidney tissue showed no change in PLDH activity (Fig. 1c).

FIG. 2.—PLDH levels in stock albinos inoculated with Moloney virus.
(a) Moloney virus as originally obtained from Dr. Moloney.
(b) Ditto after tissue culture passage.

Ordinates : Plasma lactic dehydrogenase activity (BB units $\times 10^{-3}$).
Abscissae : Days.

*Inoculation of viruses*

(a) *Moloney virus.*—The original sample obtained from Dr. Moloney, when injected into adult stock albinos, produced a rapid rise in PLDH (Fig. 2*a*). But after one, or three, passages through tissue cultures, injection produced no early change in PLDH activity (Fig. 2*b*).

In order to follow PLDH activity during leukaemogenesis by Moloney virus, newborn mice were used. Eleven stock albinos less than 24 hours old were injected with TCP virus, and their PLDH measured at intervals after weaning. No rise was detected until they were 8 weeks old, when two showed a definitely raised level. At this time all appeared healthy, but two weeks later these two, and three others, became obviously leukaemic. Thus in the development of leukaemia due to Moloney virus, as in that of transplanted leukaemia, a rise in PLDH only slightly antedates visible signs of disease.

(b) *Polyoma virus.*—Though mice which had been inoculated in infancy with polyoma virus, and bore large parotid tumours, had a high PLDH activity, injection of the virus into adult mice (with no haemaglutination-inhibiting antibodies in their serum) did not produce a rise in PLDH.

*Detection of a transmissable agent which causes a rapid rise in PLDH activity*

The fact that inoculation of S37 (the strain maintained in this laboratory) and of Moloney virus (the sample obtained from Dr. Moloney, and derived by him

FIG. 3.—PLDH activities in stock albino mice inoculated with plasma:—
(*a*) from mice bearing Sarcoma 37.
(*b*) from mice after two serial plasma passages from mice bearing Sarcoma 37.
(*c*) from mice inoculated with original Moloney virus.

Ordinates : Plasma lactic dehydrogenase activity (BB units $\times 10^{-3}$).
Abscissae : Days.

from his strain of S37) both induced a rapid rise in PLDH similar to that reported by Riley *et al.* (1960) suggested that the transmissable agent which they described was present in these inocula.

Plasma from S37-bearing mice when injected into stock albinos produced a rise of PLDH within 48 hours, and the level remained high (3000–4000 units) thereafter (Fig. 3*a*). The curve is similar to the first part of the biphasic response to inoculation of S37 tissue (i.e. before tumour growth is palpable) (Fig. 1*a*). Plasma from the inoculated mice in turn produced a similar effect on passage to other mice (Fig. 3*b* shows the 3rd passage).

Plasma of mice inoculated with the original sample of Moloney virus, when injected into other mice, also produced a rapid rise in PLDH (Fig. 3*c*).

Plasma of mice inoculated with AKR spontaneous leukaemic tissue suspension produced no change in PLDH activity of mice into which it was injected.

FIG. 4.—PLDH activities in AKR mice inoculated with

● —— ● Riley agent

○ —— ○ AKR leukaemic tissue

△ —— △ Riley agent + AKR leukaemic tissue.

Ordinates : Plasma lactic dehydrogenase activity (BB units $\times 10^{-3}$).
Abscissae : Days.

*The combined effect of inoculation of Riley agent and leukaemic cells*

An experiment was designed to discover whether the early and sustained rise in PLDH due to the transmissible virus obtained from S37 and the later rise in PLDH due to the growth of neoplastic tissue were additive phenomena or not. Of three groups of 6 AKR mice, one was inoculated with plasma from Riley agent-infected mice, one with AKR leukaemic cells, and a third with both plasma and cells. The PLDH levels of each group are shown in Fig. 4. For about the first 7 days the level in the doubly-inoculated group is approximately the sum of the levels in the other two groups, but later there is a suggestion of synergic action, for the PLDH level in the doubly-inoculated group is much higher than would be expected if the two factors were working independently.

### DISCUSSION

The biphasic rise in PLDH (Fig. 1*a*) following the inoculation of S37 is similar to that reported by Riley and Wroblewski (1960) following the injection of Ehrlich carcinoma cells. The presence in the plasma of S37-bearing mice of a transmissible agent capable of reproducing the first part of the curve is in agreement with the report of Riley *et al.* (1960), who were able to demonstrate the presence of an " enzyme-elevating " agent in the blood of mice bearing various transplanted tumours, and of eight mice with spontaneous mammary carcinomata (presence of Bittner agent not stated). However, in the present study, plasma of mice with spontaneous leukaemia, or leukaemia due to tissue culture-passaged Moloney virus infection, were not found to carry a virus of the Riley type. The original freeze-dried preparation of Moloney virus obtained from Dr. Moloney contained such an agent, but it was lost after tissue culture passage (Fig. 2*a* and 2*b*), while the leukaemogenic potency appeared actually increased by this process (Salaman, Rowson and Harvey, 1961).

It is clear from this result, as from that of injecting AKR leukaemic cells, that simple measurements of PLDH levels will not serve as an early indication of infection by leukaemogenic viruses.

Since the transplantation of a tumour inevitably involves the passage of some plasma it is not possible to say at present whether, in those cases where transplantation is followed by an early rise in PLDH, the Riley virus is actually carried in the tumour cells. Since a spontaneous leukaemia, when inoculated into mice, did not produce this early effect, it is evident that the Riley virus does not invariably accompany neoplastic tissue.

PLDH levels in AKR mice inoculated with both Riley virus and tissue suspensions from spontaneous AKR leukaemia were approximately the sum of those in mice inoculated with each separately, for the first week. Later the effect of the combined inocula rose to more than double that of the sum of the effects of each. This synergism suggests *prima facie* a linked activity. Further study of this phenomenon will include an attempt to discover the site of origin of the PLDH which each agent releases. The possibilities that the Riley agent accelerates the growth of tumours or of leukaemia, or increases the rate of loss of enzyme from neoplastic cells, will be considered.

It is a remarkable fact that PLDH level once elevated by injection of Riley virus, remains elevated for many months, and perhaps for life. No less remarkable is that the virus is demonstrable in the plasma for similar periods. Apart from their intrinsic interest these observations throw a warning light on the field of study of tumour-host relations. It is possible that some effects on the host now ascribed to tumour growth could be due to passage of viruses along with tumour inocula.

### SUMMARY

1. Inoculation into mice of the homologously transplantable tumours S37 and S180 resulted in an early rise in plasma lactic dehydrogenase (PLDH)

activity, followed by a further rise during the period of visible growth. Plasma from these mice transmitted the early effect on PLDH activity to other mice in series.

2. Inoculation into AKR mice of lymphatic tissue suspensions from spontaneous AKR leukaemia resulted in a rise in PLDH at the time of development of clinical leukaemia. In stock albino mice these suspensions produced no change in PLDH activity, and no leukaemia.

3. A preparation of Moloney virus (derived from S37 by Dr. Moloney, and obtained from him) produced an early rise in PLDH on injection into adult mice, similar to that produced by suspensions of S37.

4. Moloney virus after passage through mouse enbryo tissue cultures (TCP) had lost its power of rapidly raising PLDH, but retained its leukaemogenic activity.

5. Mice injected when newborn with TCP Moloney virus showed no rise in PLDH until shortly before the appearance of clinical leukaemia. Plasma of the leukaemic mice did not produce a rise of PLDH on injection.

6. Polyoma virus injected into polyoma-free adult mice, did not alter PLDH activity, but mice bearing large polyoma-induced parotid tumours had high PLDH levels.

7. The PLDH levels of AKR mice inoculated with both tissue suspensions from spontaneous AKR leukaemia and plasma from an S37-bearing mouse rose to more than twice the sum of the levels attained by the PLDH in mice inoculated with each separately.

8. It is concluded that :

(*a*) a transmissible agent which causes a rapid and sustained rise in PLDH is present in some homologously transplantable tumours,

(*b*) the high PLDH activity of leukaemic mice is not necessarily associated with the presence of this agent, and

(*c*) at least one tumour-producing virus (polyoma) and one leukaemogenic virus (Moloney) do not have a similar action.

Our thanks are due to Mr. L. N. Owens and Miss P. Mautner for technical assistance. The expenses for this research were partly defrayed out of a block grant from the British Empire Cancer Campaign.

### REFERENCES

Berger, L. and Broida, D.—(1960) *Tech. Bull. No.* 500 (revised) *Sigma Chemical Co.*
Hill, B. R. and Jordan, R. T.—(1957) *Cancer Res.*, **17**, 144.
*Idem* and Levi, C.—(1954) *Ibid.*, **14**, 513.
Hsieh, K. M., Suntzeff, V. and Cowdry, E. V.—(1956) *Ibid.*, **16**, 237.
Manso, C., Sugiura, F. and Wroblewski, F.—(1958) *Ibid.*, **18**, 682.
Moloney, J. B.—(1960) *J. nat. Cancer Inst.*, **24**, 933.
Riley, V., Lilly, F., Huerto, E. and Bordell, D.—(1960) *Science*, **132**, 545.
*Idem* and Wroblewski, F.—(1960) *Ibid.*, **132**, 151.
Salaman, M. H.—(1959) *Brit. J. Cancer*, **13**, 76.
*Idem* and Rowson, K. E. K.—(1959) *Rep. Brit. Emp. Cancer Campgn.*, **37**, 168.—(1960) *Laboratory Animals Centre Collected Papers*, **9**, 61.
*Iidem* and Harvey, J. J.—(1961) *Ciba Symp.* on ‘ Tumour Viruses of Murine Origin ’. In Press.
Wroblewski, F.—(1959) *Amer. J. Med.*, **27**, 911.